# Incidence and Risk Factors for 30-Day Readmission after Inpatient Chemotherapy among Acute Lymphoblastic Leukemia Patients

**DOI:** 10.3390/healthcare8040401

**Published:** 2020-10-14

**Authors:** Phuong T. Tran, William B. Slayton, Mansi Dalal, Joshua Brown

**Affiliations:** 1 Department of Pharmaceutical Outcomes and Policy, College of Pharmacy, University of Florida, Gainesville, FL 32610, USA; phuong.tran@ufl.edu; 2 Faculty of Pharmacy, Ho Chi Minh City University of Technology—HUTECH, Ho Chi Minh City 700000, Vietnam; 3 Division of Pediatric Hematology-Oncology, Department of Pediatrics, University of Florida College of Medicine, Gainesville, FL 32608, USA; slaytwb@peds.ufl.edu (W.B.S.); mdalal@peds.ufl.edu (M.D.); 4 Center for Drug Evaluation and Safety, University of Florida, Gainesville, FL 32610, USA

**Keywords:** acute lymphoblastic leukemia (ALL), acute lymphocytic leukemia, readmission, risk factors, quality of care, chemotherapy

## Abstract

Chemotherapy for acute lymphoblastic leukemia (ALL) patients is complex and intense, resulting in a high readmission rate. We aimed to identify the incidence, causes, and risk factors of readmission following inpatient chemotherapy among ALL patients, using 2016 National Readmission Database. We applied three different definitions of 30-day readmission: (1) nonelective readmission based on readmission type, (2) unplanned readmission defined by CMS, and (3) unintentional readmission, combining (1) and (2). We used unweighted multivariable Poisson regression with robust variance estimates for risk factors analysis, including patient-, hospital-, and admission-related characteristics. Percentage for nonelective, unplanned, and unintentional readmission were 33.3%, 22.4%, and 18.5%, respectively. The top three causes for unplanned readmissions were neutropenia/agranulocytosis (27.8%), septicemia (15.3%), and pancytopenia (11.5%). Risk ratios for unintentional readmission were 1.21 (1.08–1.36) for nonelective vs. elective admission, 1.19 (1.06–1.33) for public vs. private insurance enrollees, 0.96 (0.95–0.98) for each day of hospital stay, 0.77 (0.62–0.95) for large teaching and 0.87 (0.70–1.08) for small teaching vs. nonteaching hospitals. Possible strategies to reduce readmission among ALL patients could be shortening the gap in quality of care among teaching vs. non-teaching hospitals, understanding the difference between privately vs. publicly insured patients, and avoiding aggressive discharge after chemotherapy.

## 1. Introduction

Acute lymphoblastic leukemia (ALL) is a type of cancer that progresses rapidly by creating malignant white blood cells that disrupt normal blood cell production and functionality [1,2]. Based on Surveillance, Epidemiology, and End Results (SEER) data, an estimated 5930 patients were diagnosed with ALL in 2019 and the 5-year survival rate was approximately 68.6% [3]. ALL treatment is considered one of the most complex and intensive regimens in cancer therapy and generally includes five phases: induction, consolidation, interim maintenance, delayed intensification, and maintenance [4,5]. Patients may be hospitalized due to immunodeficiency, disease progression, intensive chemotherapy, or adverse effects from treatment [6].

The 30-day readmission is a popular well-accepted measurement for the quality of acute care. Readmission beyond this period is less likely due to the quality of inpatient care and rather due to patients’ behavior, disease progression, or medical care after discharge [7,8,9]. To reduce healthcare costs while providing high-quality services for patients, the Center for Medicare and Medicaid Services (CMS) uses the payment penalty for unplanned readmission and proposes penalties for unplanned hospitalization after chemotherapy. Though the ALL population is excluded from a proposed payment penalty for admission due to chemotherapy, attention should still be paid to care quality improvement in this population.

Literature regarding readmission typically uses the CMS definition of unplanned readmission, which is not applied for children with cancer. All readmissions are considered unplanned except admission for disease progression and continuation of therapy [7,8,9]. Most researchers use the CMS algorithm to define readmission. However, an emerging number of publications use the elective variable in the National Readmission Database (NRD) [10,11,12,13], leading to the concern that differing definitions generate different results and interpretations. The elective variable in NRD is defined based on readmission types reported by hospitals as either elective or non-elective (i.e., emergency or urgent).

We aimed to study the ALL national incidence, and risk factors for 30-day readmission after inpatient chemotherapy and compared three definitions, including unplanned, non-elective, and unintentional for readmission. We hypothesized that relapsed patients and patients with a longer length of stay during the index admission would be more likely to be readmitted due to disease or treatment complications. Furthermore, we hypothesized that patients treated at the teaching hospitals or high-volume hospitals would be less likely to have readmissions due to better quality of care. Lastly, a patient admitted to a hospital with an urgent or emergent index admission type would have a higher risk for readmission.

## 2. Materials and Methods

### 2.1. Data Sources

Our study was conducted using the NRD in 2016, which is the most up to date data we own to provide implications of the current clinical practice. NRD 2016 represents discharge data from 27 states, accounting for approximately 57% of all U.S. hospitalizations [14]. The data includes patients with all types of insurance and the uninsured.

NRD data are publicly available de-identified data accessed via a Data Use Agreement from the Agency for Healthcare Research and Quality.

### 2.2. Study Design and Population

**Figure 1:**
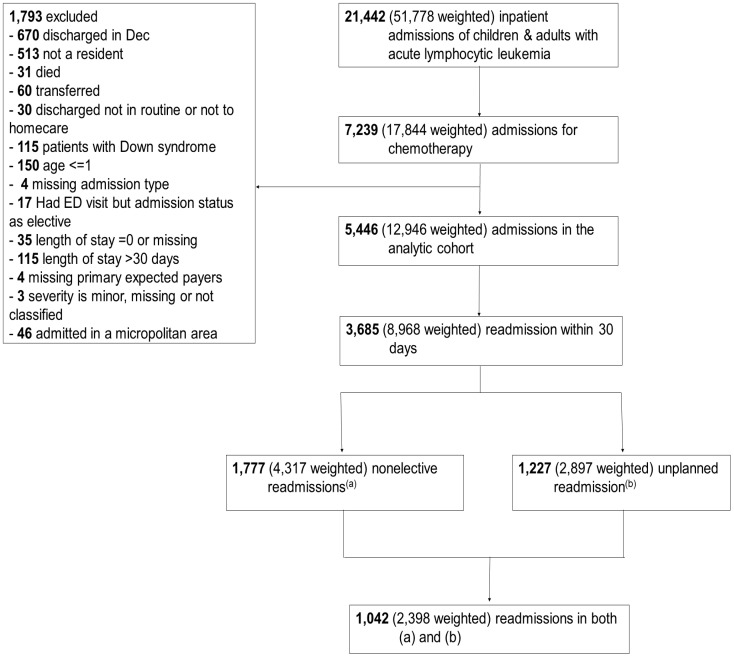
Flow diagram for study cohort inclusion.

This was a retrospective study of children and adults with ALL. We used primary and secondary International Classification of Diseases, 10th Revision, Clinical Modification (ICD-10-CM) diagnosis codes to identify patients with ALL (C91.0*). We further classified ALL states as not having achieved remission (C91.00), in remission (C91.01) or relapse (C91.02). Further, patients were required to have a primary diagnosis of an encounter for chemotherapy (Z51.11) to enter the cohort (index day). After discharge, patients were followed until readmitted or until 30 days since their index admission, whichever came first. Patients discharged in December were excluded because they did not have 30-day follow-up data to assess readmission. Patients who died or transferred to other hospitals or long-term care institutions during their index admission did not enter the study as they were no longer at risk for readmission or their readmission could not be captured. We did not include children with Down syndrome because they are often kept long-term in the hospital during their treatment to keep them from dying due to infection or adverse events. Patients with more than 30 days of hospital stay were excluded as this length of stay is unusual for the population. Patients having an elective admission resulting from an emergency visit were excluded because their reason for admission is not likely for chemotherapy. We also excluded patients one year of age or younger due to data quality and this age group patients often stay in the hospital entirely for the induction period. Because NRD does not link data across states, admissions of non-residents were also excluded. Due to a small number of records with missing expected payers, APRDRG severity, admission type, or length of stay, we omitted these missing records. Small numbers of admissions were in a micropolitan area and had minor or not classified APRDRG severity to examine their effect sizes. We, therefore, excluded them from our analysis (Figure 1). Patients could re-enter the cohort when a new admission met the inclusion and exclusion criteria.

### 2.3. Exposure

The exposure was an inpatient encounter for chemotherapy.

### 2.4. Outcome

The outcome was the first readmission within 30 days from the discharge of the index admission. We used three different definitions for the outcome.

**Definition** **1.** *A nonelective readmission was defined based on admission type (elective variable = 0) reported by hospitals in the NRD. Elective admission means the patients’ condition allowed adequate time to schedule the availability of a suitable accommodation [15]. Nonelective admission includes other admission types, such as emergency (i.e., required immediate medical intervention as a result of severe, life-threatening, or potentially disabling conditions), or urgent (required immediate attention for the care and treatment of a physical or mental disorder).*

**Definition** **2.** *An unplanned readmission was based on the algorithm developed by CMS [9,16]. Though the CMS uses the definition for adult cancer patients only, we also considered this measure for children with ALL. The CMS defines unplanned readmission as a subsequent inpatient admission occurring within 30 days of the discharge date of the index hospitalization. The readmission due to disease progression (e.g., principal diagnosis of metastatic disease) or admission for treatment (e.g., principal diagnosis of chemotherapy or radiation therapy) is not counted as readmission [16]. This definition does not include nonacute readmission in which one of 32 typically planned procedures is performed.*

**Definition** **3.** *An unintentional readmission was the combination of the two previous outcome measures.*

### 2.5. Covariables

Key covariables include age, sex, disease status, length of stay, expected payers, and hospital characteristics, such as teaching status and case volume. We categorized age into 4 categories: age 2 to ≤10; 11 to ≤18; 19 to ≤40 and >40 based on the intensity of treatment regimens for these age groups and the survival disadvantage in patients diagnosed at their 19 to ≤40 ages [17,18]. A daily charge (per 1000 USD) variable was calculated by the formula (total charge/length of stay) × 1000. Case volume was calculated as the number of ALL admissions in each hospital in 2016 (except December) and was further broken down by quintiles. We created a new variable—metropolitan hospital by combining the teaching status variable and hospital designation variable in the NRD. For severity, we used variable APR-DRGs (ALL patients refined diagnosis-related groups), which were classified as moderate, major, or extreme loss of functions based on the base APR-DRG, the severity of illness subclass, and the risk of mortality subclass. Details can be found at [19]. 

### 2.6. Statistical Analysis

The national estimate of the 30-day readmission incidence was weighted using the proc survey procedure. As missing data was minimal, we assumed that missing data occurred completely at random and excluded them from the analysis, except the total charge missing value was imputed by the median value.

To form Table 1 and Supplementary Materials Table S1 (Supplementary Materials Digital Content 1) of patient characteristics at index admission by disease status and type of admission, we used a simple frequency for binary or categorical variables and median and interquartile range (IQR) for continuous variables without weighting using ggBaseline macro [20]. Because 30-day readmission is prevalent (>10%) among the cancer population, multivariate Poisson regression with a robust variance estimate, instead of logistic regression, was used to estimate the relative risk and confidence interval [21].

Causes of readmission were classified by Clinical Classifications Software Refined (CCSR) (Healthcare Cost and utilization project—Agency for Healthcare Research and Quality, Rockville, Maryland USA) for ICD-10-CM based on the primary diagnosis code. When the primary diagnosis code was less specific and not classifiable (e.g., encounter for leukemia), we further looked at the secondary diagnosis code.

To analyze risk factors, we decided the best model was that with the most stringent criteria (unintentional readmission definition). A strict outcome measure with high specificity will provide less bias while less stringent measures with low specificity may create a bias toward the null [22]. Stratified analyses of unintentional readmission were conducted for disease status.

The analysis was conducted using SAS 9.4 (SAS Institute, Cary, NC, USA).

## 3. Results

In 2016, there was a national estimate of 51,778 admissions of ALL patients in the U.S. and 34.5% (17,844/51,778) of them admitted for chemotherapy. The weighted all-cause 30-day readmission incidence was 69.3% (8968/12,946) among eligible episodes for hospitalized chemotherapy. Weighted incidences for non-elective readmission, unplanned readmission, and unintentional readmission were 33.3% (4317/12,946), 22.4% (2897/12,946), and 18.5% (2398/12,946), respectively (Figure 1).

### 3.1. Baseline Characteristics

There were 2284 unweighted index admissions of 747 pediatric patients and 3162 unweighted index admissions of 1277 adult patients. More than half of the admissions of children (1236/2284) were classified as not having achieved remission and around 10% were in relapse (811/2284). These percentages in adults were 71.8% (2270/3162) and 13.1% (414/3162), respectively. Table 1 summarizes patients’ characteristics at the index admission by disease state (23.7% remission, 64.3% not having achieved remission, and 12.0% relapse). Adults accounted for more than 60% of patients not having achieved remission and in relapse. Relapsed patients had a higher propensity (6.0%) to be admitted on the weekend compared to patients in remission (3.1%) and patients classified as not having achieved remission (4.5%). Public and private payers accounted for approximately 95% of the study population with a similar distribution among disease states. Most of the admissions were at teaching hospitals (>90%). The number of ALL admissions in each hospital ranged from 2 to 461. The average charge was around 10,000 USD per day per patient. Supplementary Materials Table S1 gives additional insight with stratification by admission status (elective vs. nonelective). Patients with elective index admission were more likely to have elective readmission. A higher proportion of children in remission from 2 to 10 years of age were found in the nonelective (42%) vs. elective index admission group (30.4%).

### 3.2. Reasons for Readmission

Readmission after chemotherapy and immunotherapy accounted for 66.7% (2458/3685) of total readmissions. Excluding readmission for treatment continuation, the top 10 reasons for readmission were neutropenia/agranulocytosis (27.8%), septicemia (15.3%), pancytopenia (11.5%), complications of care (3.7%), disease of mouth (2.3%), fever (2.3%), coagulation and hemorrhagic disorders (2.0%), intestinal infection (1.8%), viral infection (1.8%), and pneumonia (1.5%) (Table 2). The complete causes are listed in detail in Supplementary Materials Table S2.

### 3.3. Risk Factors for 30-Day Readmission

Table 3 shows the results of three models using three different outcome definitions. We can see one variable may be a risk factor for one model but not for the other models. For example, relapsed patients were found to have an increased relative risk for readmission in unintentional (RR 1.48, 95%CI 1.20–1.81) and unplanned (RR 1.44, 95%CI 1.20–1.73) but not in non-elective (RR 1.08, 95%CI 0.93–1.24) readmission. Models of unintentional and unplanned readmission had better agreement with each other than to nonelective readmissions.

The unintentional outcome model found a statistically significant increase in readmission risk in patients classified as not having achieved remission (RR 1.28, 95%CI 1.10–1.50) and relapse (RR 1.48, 95%CI 1.20–1.81) vs. remission. Relative risk of index admission type as nonelective was 1.21 (95%CI 1.08–1.36) vs. elective. Patients with public insurance had a higher risk of readmission (RR 1.19, 95%CI 1.06–1.33) compared to those with private insurance. The readmission was lower in children aged 2–10 years (RR 0.68, 95%CI 0.58–0.81) and 11–18 years (RR 0.64, 95%CI 0.53–0.77) vs. >40 years. With each day longer for length of stay, we found a reduction in readmission risk (RR 0.97, 95%CI 0.95–0.98). We also found a reduction of risk in patients with moderate severity (RR 0.70, 95%CI 0.55–0.89) vs. extreme severity. Being admitted to a large-teaching hospital seems to be less likely admitted (RR 0.77, 95%CI 0.62–0.95) vs. nonteaching hospital.

The unplanned readmission model showed similar results to the unintentional readmission model but found a statistically significant increase in nonelective admission type (RR 1.08, 95%CI 0.97–1.20) and a statistically significant decrease in males (RR 0.90, 95%CI 0.81–0.99) vs. females. A nonelective outcome model had more discordance, including nonsignificant results in relapse (RR 1.08, 95%CI 0.93–1.24) vs. remission, ages 2–10 years (RR 0.91, 95%CI 0.81–1.01) vs. >40 years, and moderate (RR 0.89, 95%CI 0.75–1.05) vs. extreme severity. Point estimates in a non-elective readmission model tended to the null compared to the other two models.

The stratified analysis by disease states is reported in Table 4. When a patient was in relapse, none of the risk factors reached significant levels except the case volume of hospitals. Patients classified as not having achieved remission were less likely to be readmitted if they were children aged 2–10 years (RR 0.67, 95%CI 0.55–0.83), 11–18 years (RR 0.64, 95%CI 0.51–0.81), vs. >40 years. This group also had fewer readmissions with each additional day of hospital stay (RR 0.97, 95%CI 0.95–0.98) and a moderate severity of illness (RR 0.69, 95%CI 0.52–0.91) vs. extreme illness. Patients classified as not achieving remission had fewer readmissions if they were admitted to large teaching hospitals (RR 0.75, 95%CI 0.59–0.96) vs. nonteaching. Their readmission risk increased if they had a nonelective index admission (RR 1.32, 95%CI 1.15–1.51) vs. elective or had public insurance (RR 1.22, 95%CI 1.07–1.41) vs. private insurance. Risk reduction was found if patients having achieved remission included being children aged 2–10 years (RR 0.55, 95%CI 0.38–0.80), 11–18 years (RR 0.43, 95%CI 0.28–0.65) vs. >40 years, for each day longer in the length of stay (RR 0.91, 95%CI 0.86–0.96), and moderate vs. extreme severity (RR 0.32, 95%CI 0.13–0.77).

## 4. Discussion

Our U.S. nationally representative cohort included approximately 17,844 weighted chemotherapy admissions of ALL patients with a 30-day all-cause readmission rate of 69.3% and more than half of them were for treatment therapy. Readmission for nontreatment therapy was from 18.5% to 33.3% depending on outcome definitions (e.g., nonelective, unplanned, or unintentional readmission). These results are comparable with previous studies, with 30-day readmission ranging from 23% to 36% [7,23,24]. The top three reasons for readmission were neutropenia/agranulocytosis (27.8%), septicemia (15.3%), and pancytopenia (11.5%). We found treatment therapy was the most prevalent cause of readmission, which is similar to previous findings, but causes for nontreatment readmission show a difference in ranking and percentage of causes [7]. A reason could be our more diverse population, including children and adults, remission, not achieved remission, and relapse patients.

Among three definitions, we considered unintentional readmission as the most appropriate model for risk factor analysis because it is the strictest definition that combined both unplanned and nonelective readmissions. This helps to increase the specificity of the outcome measure and therefore is less likely to bias the relative risk estimate than the other two definition models [22]. However, it does not mean that the CMS unplanned readmission algorithm is less accurate. The researchers should bear in mind that the purpose of CMS algorithm is for reimbursement. Therefore, investigators should choose the most appropriate measure depending on the research question. Though three different outcome definitions—nonelective, unplanned, and unintentional readmissions—brought similar results in most risk factors, we can see that the results from the nonelective readmission model have a tendency toward the null. For example, relapsed patients had an increased relative risk for unplanned (RR 1.44, 95%CI 1.20–1.73) and unintentional (RR 1.48, 95%CI 1.20–1.81) readmission but not for non-elective (RR 1.08, 95%CI 0.93–1.24) readmissions. This tendency toward the null is true for most of our studied risk factors except admission type at index admission. This difference can be explained in that patients admitted electively for chemotherapy were also more likely to be readmitted due to other reasons. This could be an indicator of behaviors rather than risks of readmissions.

We found a longer length of stay during index admission for ALL chemotherapy resulted in fewer readmissions in patients in remission (RR 0.91, 95%CI 0.86–0.96) or patients in reinduction who had not achieved remission (RR 0.97, 95%CI 0.95–0.98) but not in newly relapsed patients (RR 0.98, 95%CI 0.96–1.00). This finding is supported by a study by Wedekind et al. [7], who showed a longer length of stay is associated with decreased risk of readmission (OR 0.44, 95%CI 0.35–0.56) vs. a short length of stay during induction therapy in newly diagnosed ALL children. In contrast, Warrick et al. [23] recommended aggressive discharge plans during induction chemotherapy for newly diagnosed high/very high risk ALL pediatric patients because of no difference in the likelihood of readmission. This discrepancy between Wedekind et al. (using a U.S. national representative sample) and Warrick et al. (using an institutional sample) could be due to the difference in the quality of care among hospitals or coding practice among hospitals. Wedekind’s and our studies both used a national representative sample, leading to our similar observations and conclusions.

Relapsed and patients classified as not having achieved remission had an increased relative risk of unintentional readmission of 1.48 (95%CI 1.20–1.81) and 1.28 (95%CI 1.10–1.50), respectively. In contrast, patients with moderate severity illness had a lower risk (RR 0.70, 95%CI 0.55–0.89) compared to extremely ill patients. This intuitively makes sense because an uncontrolled disease or extremely ill patients are more likely to be readmitted. Children aged 2–10 years (RR 0.68, 95%CI 0.58–0.81) and 11–18 years (RR 0.64, 95%CI 0.53–0.77) were less likely to be readmitted than adults >40 years. This could reflect the fact that children have a greater organ reserve than adults and the higher treatment-related toxicities of ALL adults compared to children [25]. Publicly insured patients had a higher risk (RR 1.19, 95%CI 1.06–1.33) vs. private insurance. One of the explanations could be unmeasured confounders, such as the lower social-economic status of the Medicaid population or a lack of resources in ALL treatment for patients with public insurance. Large-teaching hospitals tended to have a lower risk of readmission than non-teaching hospitals (RR 0.77, 95%CI 0.62–0.95). This may be a result of better quality of care in large teaching hospitals or better care of the emergency department in teaching hospitals, which were able to provide the medical care needed and discharge patients without requiring readmitting patients.

Our study adds to prior work in several ways. First, we used recent data (2016), which can capture the effects of the advancement in cancer therapy on readmission rates and their risk factors. Second, our nationally representative dataset is generalizable to publicly, privately, and uninsured patients. Third, the readmission rate among the ALL population is prevalent; however, most studies have reported odds ratios, which inflates the point estimate of risk when the outcome is not rare. Thus, by using Poisson regression with a robust variance estimate, our study provides risk ratio results that are often more desirable than odd ratios [21,26]. Fourth, our study analyzed several new risk factors that demonstrate a difference in the quality of care (e.g., insurance type and teaching status of hospitals). Fifth, we conducted our analyses using three different outcome definitions, which will serve to guide future studies to choose the appropriate definition for their research question. We discourage researchers from using the elective variable in NRD to define unplanned readmission because it increases measurement error and results in bias estimates toward the null. For example, one patient who is fine to receive outpatient care but chooses to be readmitted will be coded as elective but should not be considered as planned admission.

Our study also has some limitations. First, the NRD allowed us to check patients within one calendar year only, thus we may have missed readmissions of patients at the end of the calendar year. However, we excluded patients with an index admission in December to allow them to have 30 days of follow-up. Therefore, our readmission and risk factor estimates are still correct unless there is a difference between patients receiving treatment in December (e.g., holiday seasons or higher respiratory admission in winter) and other calendar months. If this is the case, then our results may not be generalizable to those who are admitted for chemotherapy in December. Second, due to the inherent limitations of the data, we were not able to adjust for all potential risk factors, such as race, a risk factor that might reflect the racial disparity on readmission among the studied population. Third, our population of interest was admitted ALL patients for chemotherapy in the U.S. Thus, the results may be not generalizable to patients with chemotherapy in outpatient settings or outside of the U.S. healthcare system. Fourth, our study is susceptible to the accuracy of the diagnosis code to define index admission and reasons for readmission. To minimize measurement errors, we limited the index admission for chemotherapy as the primary diagnosis code. When analyzing the reasons for readmission, we prioritized the primary diagnosis and used secondary diagnosis only when the primary diagnosis code could not help to categorize the cause of readmissions. Finally, claims data are built for billing but for research purposes. Thus, our results may not encompass all that was going on medically with ALL patients.

## 5. Conclusions

The top three reasons for readmission included neutropenia/agranulocytosis, septicemia, and pancytopenia, which may be reduced by efficient use of prophylactic and preventative strategies (e.g., against febrile neutropenia) and infection control protocols. Risk factors for readmission vary based on outcome definitions. Using the most stringent definition, opportunities exist to reduce readmission among ALL patients. Possible strategies to reduce readmission among ALL patients could be shortening the gap in the quality of care among teaching and nonteaching hospitals (e.g., effective and timely treatment during an emergency visit, which avoids readmissions), understanding the difference of privately vs. publicly insured patients (e.g., equitable treatment), and avoiding aggressive discharge of ALL patients after chemotherapy (e.g., safety treatment).

## Figures and Tables

**Table 1:** Unweighted characteristics of acute lymphocytic leukemia admissions in 2016.

Variables	N (%) or Median (IQR *)			
	Remission (*N* = 1289)	Not Achieved Remission (*N* = 3506)	Relapse (*N* = 651)	*p*-Value
**Age group [year]**				
[2–10]	439 (34.1)	707 (20.2)	142 (21.8)	<0.001
[11–18]	372 (28.9)	529 (15.1)	95 (14.6)	
[19–40]	238 (18.5)	821 (23.4)	207 (31.8)	
>40	240 (18.6)	1449 (41.3)	207 (31.8)	
**Admission type (elective)**	884 (68.6)	2390 (68.2)	422 (64.8)	0.200
**Readmission type**				
No readmission	415 (32.2)	1071 (30.5)	278 (42.7)	0.001
Readmission				
Non-elective	387 (44.3)	1179 (48.4)	211 (56.6)	
Elective	487 (55.7)	1256 (51.6)	162 (43.4)	
**Sex (male)**	766 (59.4)	2120 (60.5)	379 (58.2)	0.509
**Admission on a weekend**	40 (3.1)	157 (4.5)	39 (6.0)	0.010
**Primary expected payer**				
Public insurance	623 (48.3)	1691 (48.2)	331 (50.8)	0.677
Private insurance	598 (46.4)	1607 (45.8)	285 (43.8)	
Self-pay & others	68 (5.3)	208 (5.9)	35 (5.4)	
**Teaching status**				
Teaching	1179 (91.5)	3280 (93.6)	620 (95.2)	0.004
**Hospital designation**				
Large metropolitan	809 (62.8)	2524 (72.0)	499 (76.7)	<0.001
**Case volume by quintile (number ofadmissions per hospital in 2016)**				
Very high [236–461]	369 (28.6)	664 (18.9)	131 (20.1)	<0.001
High [152–235]	256 (19.9)	674 (19.2)	119 (18.3)	
Medium [96–149]	215 (16.7)	703 (20.1)	169 (26.0)	
Low [53–94]	287 (22.3)	652 (18.6)	133 (20.4)	
Very low [2–52]	162 (12.6)	813 (23.2)	99 (15.2)	
**Length of stay**				
Median (IQR *)	4.0 (3.0–4.0)	4.0 (3.0–5.0)	5.0 (3.0–10.0)	<0.001
Daily charge (1000 USD **)				
Median (IQR *)	8.6 (5.6–12.3)	9.3 (6.3–13.4)	11.2 (7.0–17.6)	<0.001

(*) interquartile range, (**) Unitted States Dollar.

**Table 2:** Causes of unplanned readmissions based on Clinical Classifications Software Refined.

Causes of Readmission Based on Clinical Classifications Software Refined	*N* = 1227
Diseases of white blood cells (e.g., neutropenia, agranulocytosis)	27.8%
Septicemia	15.3%
Pancytopenia	11.5%
Complications of other surgical or medical care, injury	3.7%
Diseases of mouth; excluding dental	2.3%
Fever	2.3%
Coagulation and hemorrhagic disorders	2.0%
Intestinal infection	1.8%
Viral infection	1.8%
Pneumonia (except that caused by tuberculosis)	1.5%
Others	30.0%

**Table 3:** The relative risk of factors influencing all-cause 30-day readmission of patients with acute lymphocytic leukemia.

	Risk Ratio (95% Confidence Interval)		
*N* = 5446	Unintentional Readmission (*N* = 1042)	Unplanned Readmission (*N* = 1227)	Non-Elective Readmission (*N* = 1777)
**Disease state (ref remission)**			
Not achieved remission	1.28 (1.10–1.50) *	1.24(1.08–1.42) *	1.12 (1.01–1.23) *
Relapse	1.48 (1.20–1.81) *	1.44 (1.20–1.73) *	1.08 (0.93–1.24)
**Age group (ref >40 age)**			
[2–10]	0.68 (0.58–0.81) *	0.80 (0.69–0.92) *	0.91 (0.81–1.01)
[11–18]	0.64 (0.53–0.77) *	0.73 (0.62–0.86) *	0.88 (0.78–0.99) *
[19–40]	0.90 (0.79–1.04)	1.00 (0.88–1.13)	0.96 (0.87–1.06)
**Admission type (ref elective)**			
Nonelective	1.21 (1.08–1.36) *	1.08 (0.97–1.20)	2.05 (1.90–2.22) *
**Length of stay (days)**	0.96 (0.95–0.98) *	0.97 (0.95–0.98) *	0.98 (0.97–0.99) *
**Severity (ref extreme)**			
Moderate	0.70 (0.55–0.89) *	0.69 (0.56–0.86) *	0.89 (0.75–1.05)
Major	0.83 (0.66–1.04)	0.84 (0.69–1.02)	0.95 (0.81–1.12)
**Primary Payers (ref private insurance)**			
Public insurance	1.19 (1.06–1.33) *	1.18 (1.06–1.30) *	1.10 (1.02–1.19) *
Self-pay & others	1.07 (0.84–1.37)	1.04 (0.83–1.29)	1.07 (0.91–1.25)
Sex (ref female)			
Male	0.89 (0.80–1.00)	0.90 (0.81–0.99) *	0.97 (0.90–1.04)
**Metropolitan hospital (ref nonteaching)**			
Large, teaching	0.77 (0.62–0.95) *	0.82 (0.67–0.99) *	0.85 (0.74–0.99) *
Small, teaching	0.87 (0.70–1.08)	0.93 (0.77–1.14)	1.05 (0.90–1.21)
**Case volume (ref very low case volume)**			
Very high	1.13 (0.94–1.36)	1.01 (0.85–1.20)	0.99 (0.87–1.12)
High	0.97 (0.81–1.18)	0.87 (0.74–1.04)	0.81 (0.71–0.93) *
Medium	0.98 (0.82–1.18)	0.98 (0.84–1.15)	0.94 (0.83–1.06)
Low	1.10 (0.92–1.31)	1.03 (0.88–1.20)	1.03 (0.93–1.16)

* confidence intervals do not cross 1.

**Table 4:** The relative risk of factors influencing all-cause 30-day unintentional readmission of patients with acute lymphocytic leukemia stratifyied by disease states.

	Risk Ratio (95% Confidence Interval)		
	Remission (*N* = 1289)	Not Achieved Remission (*N* = 3506)	Relapse (*N* = 651)
**Age group (ref >40 age)**			
[2–10]	0.55 (0.38–0.80) *	0.67 (0.55–0.83) *	0.85 (0.56–1.27)
[11–18]	0.43 (0.28–0.65) *	0.64 (0.51–0.81) *	1.13 (0.74–1.72)
[19–40]	0.72 (0.48–1.08)	0.92 (0.78–1.09)	1.02 (0.71–1.46)
**Admission type (ref elective)**			
Non-elective	1.16 (0.85–1.58)	1.32 (1.15–1.51) *	0.92 (0.68–1.24)
**Length of stay (days)**	0.91 (0.86–0.96) *	0.97 (0.95–0.98) *	0.98 (0.96–1.00)
**Severity (ref extreme)**			
Moderate	0.32 (0.13–0.77) *	0.69 (0.52–0.91) *	0.96 (0.57–1.62)
Major	0.45 (0.19–1.07)	0.78 (0.60–1.02)	1.10 (0.71–1.72)
**Primary Payers (ref private insurance)**			
Public insurance	1.03 (0.78–1.38)	1.22 (1.07–1.41) *	1.10 (0.83–1.47)
Self-pay and others	1.16 (0.66–2.07)	1.05 (0.79–1.40)	0.90 (0.45–1.82)
**Sex (ref female)**			
Male	0.87 (0.66–1.14)	0.90 (0.79–1.03)	0.90 (0.68–1.19)
**Metropolitan hospital (ref nonteaching)**			
Large, teaching	0.75 (0.46–1.23)	0.75 (0.59–0.96) *	1.53 (0.49–4.69)
Small, teaching	0.84 (0.52–1.37)	0.81 (0.62–1.04)	2.18 (0.70–6.77)
**Case volume (ref very low case volume)**			
Very high	1.10 (0.68–1.79)	1.09 (0.87–1.36)	2.17 (1.19–3.97) *
High	1.10 (0.66–1.83)	0.93 (0.75–1.16)	1.56 (0.82–2.96)
Medium	1.02 (0.61–1.72)	0.90 (0.72–1.12)	1.87 (1.03–3.38) *
Low	1.23 (0.78–1.93)	1.00 (0.82–1.24)	2.09 (1.15–3.81) *

* confidence intervals do not cross 1.

## Supplementary Materials

The following are available online at https://www.mdpi.com/2227-9032/8/4/401/s1, Table S1: Unweighted characteristics of acute lymphocytic leukemia admission in 2016, Table S2: Complete causes of readmission based on Clinical Classifications Software Refined (CCSR).
