# Supplementary material for: Incidence and Risk Factors for 30-Day Readmission after Inpatient Chemotherapy among Acute Lymphoblastic Leukemia Patients
Source: Healthcare (Basel). 2020 Oct 14;8(4):401. doi: 10.3390/healthcare8040401 (PMC7720128; doi:10.3390/healthcare8040401)
Supplement: Supplementary file 1 [file healthcare-08-00401-s001.zip › healthcare-920667-supplementary.docx]

**Supplemental Materials**

**Table S1. Unweighted characteristics of acute lymphocytic leukemia admission in 2016**

| N (%) or Median (IQR*) | | | | | | |  |
| --- | --- | --- | --- | --- | --- | --- | --- |
|  | Remission | | Not achieved remission | | Relapse | |  |
| Variables | Elective (N=884) | Non-elective  (N=405) | Elective (N=2390) | Non-elective  (N=1116) | Elective (N=422) | Non-elective  (N=229) | P Value |
| **Age group (year)** |  |  |  |  |  |  |  |
| [2-10] | 269 (30.4) | 170 (42.0) | 493 (20.6) | 214 (19.2) | 95 (22.5) | 47 (20.5) | <.001 |
| [11-18] | 257 (29.1) | 115 (28.4) | 368 (15.4) | 161 (14.4) | 59 (14.0) | 36 (15.7) |  |
| [19-40] | 169 (19.1) | 69 (17.0) | 539 (22.6) | 282 (25.3) | 141 (33.4) | 66 (28.8) |  |
| >40 | 189 (21.4) | 51 (12.6) | 990 (41.4) | 459 (41.1) | 127 (30.1) | 80 (34.9) |  |
| **Readmission type** |  |  |  |  |  |  |  |
| No readmission | 286 (32.4) | 129 (31.9) | 753 (31.5) | 318 (28.5) | 171 (40.5) | 107 (46.7) | <.001 |
| Readmission |  |  |  |  |  |  |  |
| Non-elective | 178 (29.8) | 209 (75.7) | 588 (35.9) | 591 (74.1) | 121 (48.2) | 90 (73.8) |  |
| Elective | 420 (70.2) | 67 (24.3) | 1049 (64.1) | 207 (25.9) | 130 (51.8) | 32 (26.2) |  |
| **Sex (male)** | 502 (56.8) | 264 (65.2) | 1446 (60.5) | 674 (60.4) | 252 (59.7) | 127 (55.5) | 0.059 |
| **Admission at weekend** | 28 (3.2) | 12 (3.0) | 101 (4.2) | 56 (5.0) | 28 (6.6) | 11 (4.8) | 0.041 |
| **Primary expected payer** |  |  |  |  |  |  |  |
| Public insurance | 412 (46.6) | 211 (52.1) | 1143 (47.8) | 548 (49.1) | 198 (46.9) | 133 (58.1) | 0.060 |
| Private insurance | 422 (47.7) | 176 (43.5) | 1116 (46.7) | 491 (44.0) | 198 (46.9) | 87 (38.0) |  |
| Self-pay & others | 50 (5.7) | 18 (4.4) | 131 (5.5) | 77 (6.9) | 26 (6.2) | 9 (3.9) |  |
| **Teaching status** |  |  |  |  |  |  |  |
| Teaching | 805 (91.1) | 374 (92.3) | 2266 (94.8) | 1014 (90.9) | 398 (94.3) | 222 (96.9) | <.001 |
| **Hospital designation** |  |  |  |  |  |  |  |
| Large metropolitan | 641 (72.5) | 168 (41.5) | 1796 (75.1) | 728 (65.2) | 330 (78.2) | 169 (73.8) | <.001 |
| **Case volume (quintile)** |  |  |  |  |  |  |  |
| Very high [236-461] | 244 (27.6) | 125 (30.9) | 548 (22.9) | 116 (10.4) | 96 (22.7) | 35 (15.3) | <.001 |
| High [152-235] | 216 (24.4) | 40 (9.9) | 500 (20.9) | 174 (15.6) | 74 (17.5) | 45 (19.7) |  |
| Medium [96-149] | 148 (16.7) | 67 (16.5) | 431 (18.0) | 272 (24.4) | 109 (25.8) | 60 (26.2) |  |
| Low [53-94] | 164 (18.6) | 123 (30.4) | 441 (18.5) | 211 (18.9) | 88 (20.9) | 45 (19.7) |  |
| Very low [2-52] | 112 (12.7) | 50 (12.3) | 470 (19.7) | 343 (30.7) | 55 (13.0) | 44 (19.2) |  |
| **Length of stay** |  |  |  |  |  |  |  |
| Median (IQR)* | 4.0 (3.0–5.0) | 4.0 (3.0–4.0) | 4.0 (3.0–5.0) | 4.0 (3.0–6.0) | 4.0 (3.0–10.0) | 5.0 (3.0–11.0) | <.001 |
| **Daily charge (1000 USD)** |  |  |  |  |  |  |  |
| Median (IQR)* | 9.0 (5.8–13.0) | 7.6 (5.1–10.2) | 9.4 (6.4–13.3) | 9.1 (6.0–13.6) | 11.9 (7.1–18.3) | 10.4 (6.9–15.8) | <.001 |

(*) interquartile range

Table S2. Complete causes of readmission based on Clinical Classifications Software Refined (CCSR)

| Causes of readmission based on Clinical Classifications Software Refined (CCSR) |  |
| --- | --- |
| Diseases of white blood cells | 27.8% |
| Septicemia | 15.3% |
| Pancytopenia | 11.5% |
| Complication of other surgical or medical care, injury | 3.7% |
| Diseases of mouth; excluding dental | 2.3% |
| Fever | 2.3% |
| Coagulation and hemorrhagic disorders | 2.0% |
| Intestinal infection | 1.8% |
| Viral infection | 1.8% |
| Pneumonia (except that caused by tuberculosis) | 1.5% |
| Fluid and electrolyte disorders | 1.3% |
| Skin and subcutaneous tissue infections | 1.2% |
| Acute and unspecified renal failure | 1.2% |
| Abnormal findings without diagnosis | 0.9% |
| Noninfectious gastroenteritis | 0.9% |
| Complication of cardiovascular device, implant or graft, initial encounter | 0.8% |
| Drug induced or toxic related condition | 0.8% |
| Epilepsy; convulsions | 0.8% |
| Urinary tract infections | 0.8% |
| Adverse effects of medical drugs | 0.7% |
| Other specified and unspecified gastrointestinal disorders | 0.7% |
| Pancreatic disorders (excluding diabetes) | 0.7% |
| Abdominal pain and other digestive/abdomen signs and symptoms | 0.6% |
| Essential hypertension | 0.6% |
| Malnutrition | 0.6% |
| Nausea and vomiting | 0.6% |
| Pleurisy, pleural effusion and pulmonary collapse | 0.6% |
| Appendicitis and other appendiceal conditions | 0.5% |
| Immunity disorders | 0.5% |
| Gastrointestinal and biliary perforation | 0.5% |
| Other specified nervous system disorders | 0.5% |
| Acute pulmonary embolism | 0.4% |
| Gastritis and duodenitis | 0.4% |
| Other specified upper respiratory infections | 0.4% |
| Postprocedural or postoperative digestive system complication | 0.4% |
| Respiratory signs and symptoms | 0.4% |
| Acute phlebitis; thrombophlebitis and thromboembolism | 0.3% |
| Allergic reactions | 0.3% |
| Anal and rectal conditions | 0.3% |
| External cause codes: poisoning by drug | 0.3% |
| Influenza | 0.3% |
| Malaise and fatigue | 0.3% |
| Musculoskeletal pain | 0.3% |
| Other specified and unspecified nutritional and metabolic disorders | 0.3% |
| Paralysis (other than cerebral palsy) | 0.3% |
| Acute hemorrhagic cerebrovascular disease | 0.2% |
| Biliary tract disease | 0.2% |
| Diabetes mellitus with complication | 0.2% |
| Fungal infections | 0.2% |
| Intestinal obstruction and ileus | 0.2% |
| Nervous system signs and symptoms | 0.2% |
| Obesity | 0.2% |
| Other general signs and symptoms | 0.2% |
| Other specified and unspecified liver disease | 0.2% |
| Respiratory failure; insufficiency; arrest | 0.2% |
| Acute posthemorrhagic anemia | 0.2% |
| Alcohol-related disorders | 0.2% |
| Bacterial infections | 0.2% |
| Chronic phlebitis; thrombophlebitis and thromboembolism | 0.2% |
| Complication of transplanted organs or tissue, initial encounter | 0.2% |
| Conditions due to neoplasm or the treatment of neoplasm | 0.2% |
| Diverticulosis and diverticulitis | 0.2% |
| Drug induced or toxic related condition | 0.2% |
| Headache; including migraine | 0.2% |
| Hemorrhoids | 0.2% |
| Hypertension with complications and secondary hypertension | 0.2% |
| Meningitis | 0.2% |
| Muscle disorders | 0.2% |
| Myocarditis and cardiomyopathy | 0.2% |
| Other specified and unspecified disorders of the ear | 0.2% |
| Other specified connective tissue disease | 0.2% |
| Sinusitis | 0.2% |
| Skin/Subcutaneous signs and symptoms | 0.2% |
| Stress fracture, initial encounter | 0.2% |
| Traumatic brain injury (TBI); concussion, initial encounter | 0.2% |
| Acute bronchitis | 0.1% |
| Aspiration pneumonitis | 0.1% |
| Asthma | 0.1% |
| Benign neoplasms | 0.1% |
| Cardiac dysrhythmias | 0.1% |
| Cardiac dysrhythmias | 0.1% |
| Chronic obstructive pulmonary disease and bronchiectasis | 0.1% |
| Circulatory signs and symptoms | 0.1% |
| CNS abscess | 0.1% |
| Complication of internal orthopedic device or implant | 0.1% |
| Coronary atherosclerosis and other heart disease | 0.1% |
| Depressive disorders | 0.1% |
| Drug induced or toxic related condition | 0.1% |
| Esophageal disorders | 0.1% |
| Fracture of the upper limb | 0.1% |
| Heart failure | 0.1% |
| Hemolytic anemia | 0.1% |
| Hepatitis | 0.1% |
| Hypotension | 0.1% |
| Inflammatory conditions of male genital organs | 0.1% |
| Myopathies | 0.1% |
| Neuro-ophthalmology | 0.1% |
| Nonspecific chest pain | 0.1% |
| Nutritional deficiencies | 0.1% |
| Occlusion or stenosis of precerebral or cerebral arteries without infarction | 0.1% |
| Open wounds of head and neck, initial encounter | 0.1% |
| Opioid-related disorders | 0.1% |
| Osteoporosis | 0.1% |
| Other specified and unspecified diseases of bladder and urethra | 0.1% |
| Other specified and unspecified endocrine disorders | 0.1% |
| Other specified and unspecified hematologic conditions | 0.1% |
| Other specified and unspecified skin disorders | 0.1% |
| Other specified status | 0.1% |
| Otitis media | 0.1% |
| Pericarditis and pericardial disease | 0.1% |
| Peripheral and visceral vascular disease | 0.1% |
| Personal/family history of disease | 0.1% |
| Pituitary disorders | 0.1% |
| Pneumothorax | 0.1% |
| Polyneuropathies | 0.1% |
| Retinal and vitreous conditions | 0.1% |
| Shock | 0.1% |
| Socioeconomic/psychosocial factors | 0.1% |
| Superficial injury; contusion, initial encounter | 0.1% |
| Symptoms of mental and substance use conditions | 0.1% |
| Syncope | 0.1% |
| Trauma- and stressor-related disorders | 0.1% |
| Urinary incontinence | 0.1% |
